# RPS7 inhibits colorectal cancer growth via decreasing HIF-1α-mediated glycolysis

**DOI:** 10.18632/oncotarget.6807

**Published:** 2015-12-31

**Authors:** Wen Zhang, Duo Tong, Fei Liu, Dawei Li, Jiajia Li, Xi Cheng, Ziliang Wang

**Affiliations:** ^1^ Cancer Institute and Department of Medical Oncology, Fudan University Shanghai Cancer Center, Shanghai 200032, China; ^2^ Department of Gynecological Oncology, Fudan University Shanghai Cancer Center, Shanghai 200032, China; ^3^ Department of Colorectal Surgery, Fudan University Shanghai Cancer Center, Shanghai 200032, China; ^4^ Department of Oncology, Shanghai Medical College, Fudan University, Shanghai 200032, China

**Keywords:** RPS7, HIF-1α, glycolysis, proliferation, CRC

## Abstract

Ribosomal protein S7 (RPS7) acts as a tumor suppressor in primary tumorigenesis but its role in cancer metabolism remains unclear. In this study, we demonstrate that RPS7 inhibits the colorectal cancer (CRC) cell glycolysis by suppressing the expression of hypoxia-inducible transcription factor-1α (HIF-1α) and the metabolic promoting proteins glucose transporter 4 (GLUT4) and lactate dehydrogenase B (LDHB). Further study found that the enhanced expression of HIF-1α abrogates the overexpression effects of RPS7 on CRC. *In vivo* assays also demonstrate that RPS7 suppresses colorectal cancer tumorigenesis and glycolysis. Clinically, the tissue microarray (TMA) analysis discloses the negative regulatory association between RPS7 and HIF-1α in colorectal cancer. Meanwhile, overexpression of RPS7 in colorectal cancer tissues predicts good overall survival and progression-free survival, but high expression level of HIF-1α indicates poor overall survival and progression-free survival. Overall, we reveal that RPS7 inhibits colorectal cancer glycolysis through HIF-1α-associated signaling and may be a promising biomarker for prognosis prediction and a potential target for therapeutic treatment.

## INTRODUCTION

Colorectal cancer (CRC) is one of the most common cancers worldwide, ranking the third of the leading causes of cancer death [1]. For patients with early stage, the 5-year relative survival rate is about 90%, however, the 5-year relative survival rate drops to 10% for patients with advanced disease [2]. Traditional TNM (T, tumor; N, lymph node; M, metastasis) staging system provides important information for predicting prognosis, while even with the same stage some CRC show worse biological behaviors. So it's of great importance to find proper biomarkers to figure out this part of CRC patients and choose personalized treatment and follow-up plan.

Ribosomal proteins (RPs) are main components of ribosomes [3], which are essential in the process of translation and crucial for ribosome assembly [4]. Growing evidences show that the abnormalities in RPs may lead to anemia [5] and tumorigenesis [6, 7]. Some RPs promote the process of oncogenesis, such as silencing of RPS13 restrains the growth of gastric cancer [8] and down-regulation of RPL6 inhibits the proliferation of gastric cancer [9]. On the other hand, certain RPs, such as RPL41, RPL5 and RPL11, are considered to be cancer suppressors [10–12]. Otherwise, parts of RPs have the potential to be prognostic or diagnostic markers in various cancers, for instance increased RPL13 expression has been found to be positively correlated with clinical staging of gastric cancers [13], and low expression of RPS4 is associated with poor prognosis in ovarian cancer [14]. However, the exact role of RPs in cancers and their underlying mechanisms still remain largely unknown.

Ribosomal small subunit protein 7(RPS7) is an important component of the small subunit of ribosomes. The mutations of RPS7 have been shown to be associated with Diamond-Blackfan Anemia (DBA) [5]. In zebrafish, RPS7 has been found to regulate cell apoptosis and cell cycle progression by p53, in spite of affecting the process of hematopoiesis [15]. Further research indicated that RPS7 might serve as a novel MDM2-interacting partner to be involved in the MDM2-P53 interaction and stabilize P53 protein [16, 17]. In addition, our previous study observed that the RPS7 might works as a tumor suppressor in ovarian cancer through PI3K/AKT and MAPK signal networks [18]. Nevertheless, the regulatory role of RPS7 in colorectal tumorigenesis still needs further study.

One important hallmark of cancer is the energy metabolism reprogram [19]. In 1920s Warburg proposed the “Warburg phenomenon” consisting of an increase in glycolysis and the enhanced lactate production [20, 21]. The altered metabolism confers cancer cells a unique tumor microenvironment and provides cells with intermediates needed for biosynthetic pathways [22, 23]. As tumor expands, it outgrows beyond the supplying district of its local blood, leading to hypoxia and activating hypoxia-inducible transcription factor 1(HIF-1) [24]. HIF-1 is composed of two subunits, the inducibly expressed HIF-1α and the constitutively expressed HIF-1β [25]. HIF-1 stimulates cells to shift toward glycolysis and produce lactate to adapt to hypoxia [26]. The activation of HIF-1α promotes tumor progression [27, 28]. And HIF-1α also plays a role in the regulation of MDM2-P53 pathway [29].

In this study, we found that the overexpression of RPS7 inhibited, whereas the silencing of RPS7 promoted, the growth, proliferation and glycolysis of CRC cells both *in vitro* and *in vivo*. We further demonstrated that RPS7 interacted with HIF-1α and negatively regulated the expression of HIF-1α. Our results suggest that RPS7 plays as a tumor suppressor to inhibit the growth and glycolysis of CRC by suppressing HIF-1α.

## RESULTS

### RPS7 inhibits CRC cell proliferation

**Figure 1:**
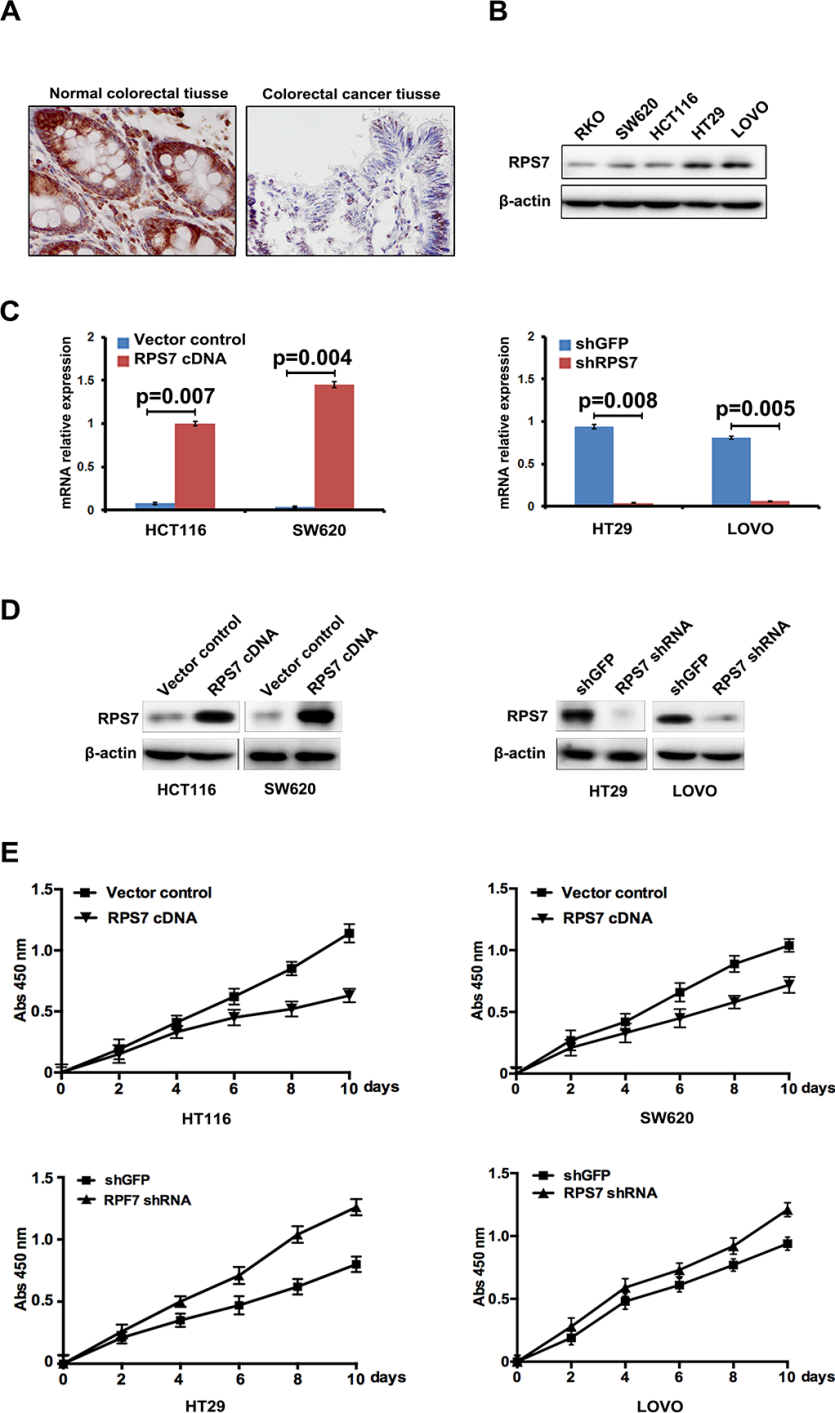
Expression of RPS7 in normal colorectal tissues, CRC tissues and CRC cell lines and the effect of RPS7 on CRC cell proliferation (**A**) Detection of expression level of RPS7 in colorectal normal and cancer tissues by IHC. (**B**) Detection of RPS7 by Western blotting in RKO, SW620, HCT116, HT29 and LOVO cells. (**C**–**D**) Analyses of RPS7 by realtime-PCR (C) and Western blotting (D) in cell expressing RPS7 cDNA or shRNA and the corresponding control plasmids. (**E**) Detection of cell proliferation by CCK8 (*P* < 0.05). Error bars = 95% confidence intervals (CIs).

Firstly, we detected the expression level of RPS7 in human normal colorectal tissues and colorectal cancer tissues, founding that the expression level of RPS7 in the normal colorectal tissues was higher than that in colorectal cancer tissues (Figure 1A), indicating that RPS7 may be a tumor suppressor in colorectal cancer. To investigate the function of RPS7 in CRC, we further detected the expression level of RPS7 in five human CRC cell lines, including RKO, SW620, HCT116, HT29 and LOVO cells. We found that the expression levels of RPS7 in LOVO and HT29 cells were high, but low in in HCT116, RKO and SW620 cells (Figure 1B). So, we established SW620 and HCT116 cells stably expressing RPS7 cDNA by inserting RPS7 cDNA into cells to obtain SW620/ RPS7 cDNA and HCT116/RPS7 cDNA cells (corresponding control cells were infected with vector control). We introduced retroviruses carrying shRNA against RPS7 into HT29 and LOVO cells to generate HT29/RPS7 shRNA LOVO/RPS7 shRNA cells (corresponding control cells were infected with retroviruses expressing shGFP). The results of real-time PCR and Western blotting confirmed that the mRNA level and the protein level of RPS7 were both up-regulated in SW620/ RPS7 cDNA and HCT116/ RPS7 cDNA cells and both down-regulated in HT29/ RPS7 shRNA and LOVO/ RPS7 shRNA cells, compared with their corresponding controls (Figure 1C and 1D).

We next evaluated the effect of RPS7 on the proliferation of CRC cells by CCK-8 assay. The results showed that SW620/ RPS7 cDNA and HCT116/RPS7 cDNA cells had lower level of proliferation than their corresponding control cells (Figure 1E), and HT29/ RPS7 shRNA and LOVO/ RPS7 shRNA cells had higher level of proliferation than corresponding control cells expressing shGFP (Figure 1E), which demonstrated that RPS7 could inhibit the proliferation of CRC cells as a tumor suppressor.

### RPS7 inhibits the glucose metabolism and lactate production in CRC cells

**Figure 2:**
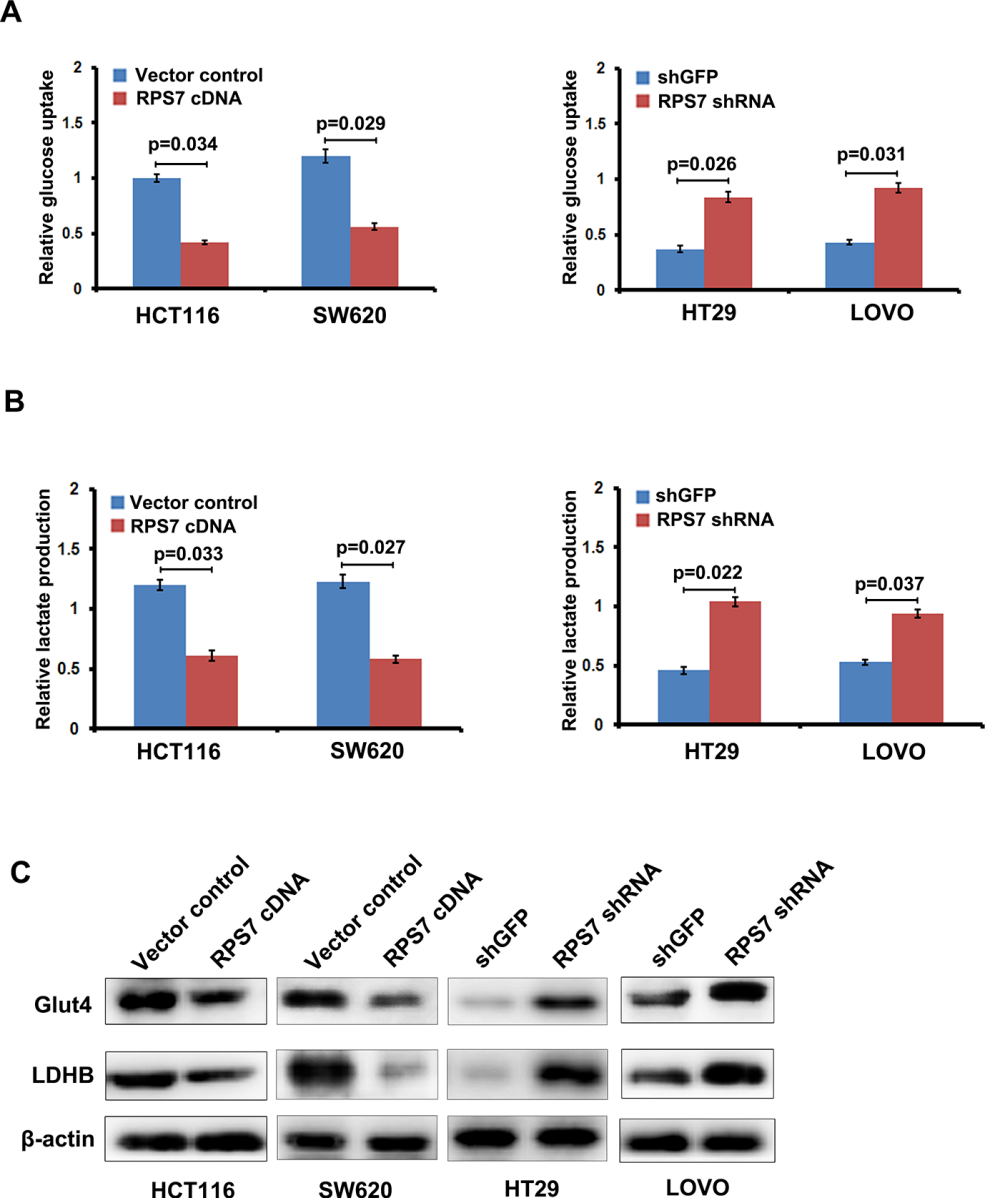
RPS7 inhibits glycolysis in colorectal cancer cells (**A**) Effect of RPS7 overexpression and knock-down on glucose uptake capacity of colorectal cancer cells. (**B**) Effect of RPS7 overexpression and knock-down on lactate production of colorectal cancer cells. (**C**) Effect of RPS7 overexpression and knock-down on the expression level of key glycolytic enzymes.

Previous studies showed that tumor formation and progression require glucose metabolism accordingly. Under some stress, such as hypoxic stress, cancer cells exhibit a shift of glucose metabolism to less efficient glycolytic pathways. To confirm whether changed viability and tumorigenic potential of the colorectal cells expressing RPS7 cDNA or shRNA were due to the change in glycolysis, we performed glucose uptake and lactate production assay. We found that overexpression of RPS7 strongly abrogated, whereas silencing of RPS7 promoted the ability of CRC cells to take in glucose and produce lactate, suggesting that RPS7 is involved in glucose metabolism in CRC cells (Figure 2A and 2B). We further investigated the effect of RPS7 on the expression level of metabolism-associated proteins in CRC cells by western blotting. As shown in Figure 2C, the up-regulation of RPS7 remarkably decreased, while down-regulation of RPS7 increased the expression level of GLUT4 and LDHB. These results demonstrated that RPS7 have the function of inhibiting the glycolysis in the colorectal cancer cells by down-regulating GLUT4 and LDHB, which further changed the viability and tumorigenic potential of colorectal cancer cells.

### RPS7 down-regulates HIF-1α in CRC cells *in vitro*

**Figure 3:**
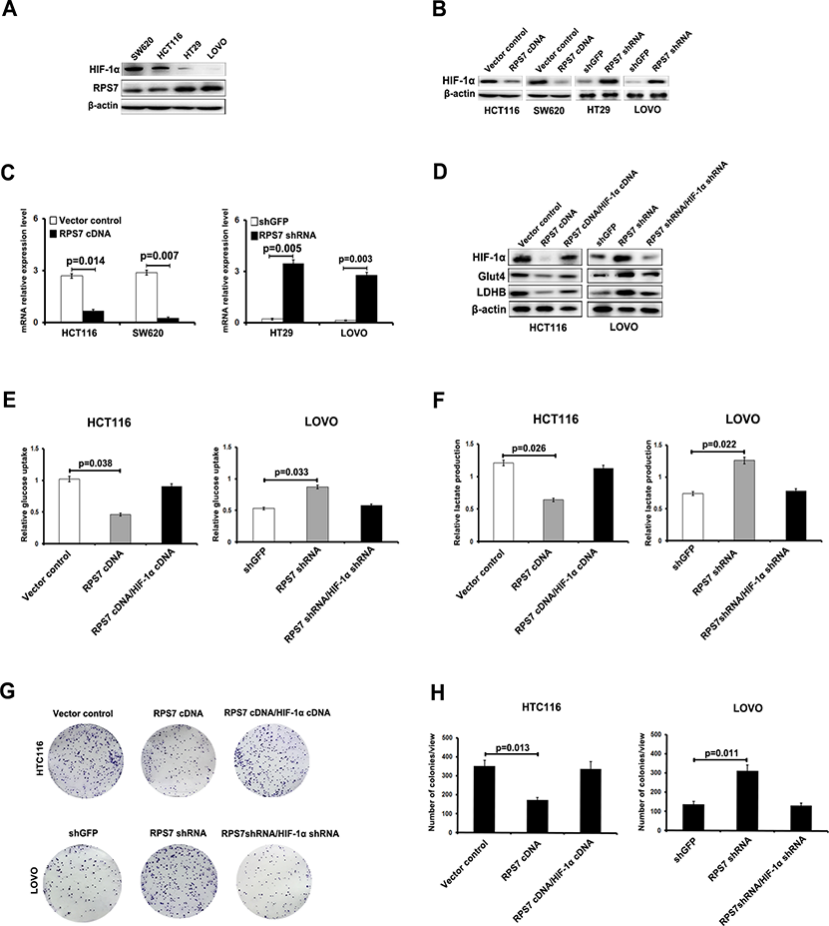
RPS7 interacts with HIF-1α to regulate the glucose uptake capacity and lactate production of colorectal cancer cells (**A**) The expression level of HIF-1α is detected by Western blotting in SW620, HCT116, HT29 and LOVO cells. (**B**–**C**) Detection of expression in RPS7-overexpressing by western blot and realtime PCR, RPS7-silenced cells and corresponding control cells treated with Cocl2 (300 μM). (**D**) HIF-1α rescues the inhibition of RPS7on GLUT4 and LDHB. (**E**–**F**) HIF-1α rescues the effect of RPS7 on glucose uptake capacity and lactate production of colorectal cancer cells. (**G**–**H**) HIF-1α rescues the effect of RPS7 on the colony formation ability of colorectal cancer cells.

The hypoxia-inducible factor-1α (HIF-1α) primarily senses and adapts cells to changes in the O_2_ level and maintains cell viability under hypoxic stress conditions. HIF-1α is found to mediate the cell metabolism transformation. Studies showed that GLUT4 was HIF-1α-responsive genes [30, 31], so we inferred that RPS7 might interact with HIF-1α to regulate the expression level of GLUT4 or LDHB. We firstly detected the expression level of HIF-1α in the CRC cell lines, including SW620, HCT116, HT29 and LOVO cells. We found that the expression level of HIF-1α was lower in LOVO and HT29 cells, but higher in HCT116 and SW620 cells, which was contrary to the expression level of RPS7 (Figure 3A). In the presence of CoCl_2_, which induces HIF-1α protein level stability, we detected the expression level of HIF-1α in HCT116/ RPS7 cDNA and SW620/ RPS7 cDNA cells, and HT29/ RPS7 shRNA and LOVO/ RPS7 shRNA cells. We found that the expression level of HIF-1α, detected by realtime-PCR and western blot, increased in RPS7-silenced CRC cells but decreased in RPS7-overexpressing CRC cells, compared with the corresponding control cells, suggesting that RPS7 negatively regulated the expression of HIF-1α in CRC cells (Figure 3B and 3C). Meanwhile, we found that the induction of HIF-1α cDNA into HCT116/ cDNA cells or HIF-1α shRNA into LOVO/ RPS7 shRNA cells could rescue the expression level of GLUT4 and LDHB in HCT116/ cDNA cells or LOVO/ RPS7 shRNA cells respectively (Figure 3D). We further performed the glucose uptake and lactate production, and colony formation assays to demonstrate the anti-RPS7 effect of HIF-1α on the growth of colorectal cancer cells. The results showed that HIF-1α recovered the metabolic status (Figure 3E and 3F) and colony formation ability (Figure 3G and 3H) of RPS7-upregulated and RPS7-downregulated cells, compared with corresponding control cells. The above results indicated that the anti-metabolic effects of RPS7 in CRC cells might be achieved by down-regulating HIF-1α.

### RPS7 inhibits proliferation and glucose metabolism of CRC cells *in vivo*

**Figure 4:**
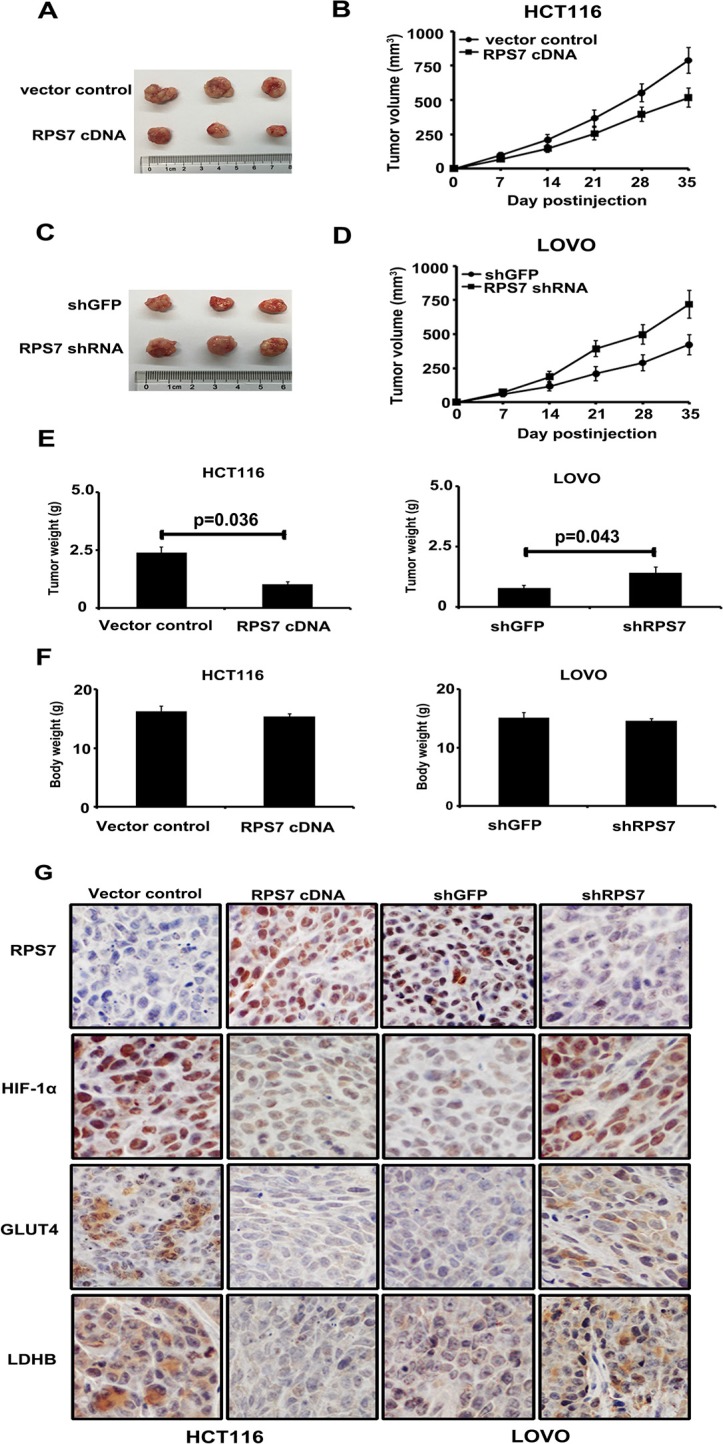
Xenograft tumor burden in mice with overexpression or silencing of RPS7 (**A**–**D**) *In vivo* tumorigenesis examined by animal assay and subcutaneous tumor growth from mice injected with cells expressing either RPS7 cDNA or shRNA and the corresponding controls (*P* < 0.05). Error bars = 95% CIs. (**E**–**F**) Figures show tumor and body weights of mice at the end of observation. (**G**) Immunohistochemical staining of xenograft tumor tissues. Tissues were stained with rabbit anti-RPS7, HIF-1α, GLUT4 and LDHB antibodies and visualized with donkey anti-mouse secondary antibody (Magnification × 400).

The effect of RPS7 on the CRC metabolism *in vivo* was investigated by injecting HCT116/ RPS7 cDNA cells or LOVO/ RPS7 shRNA cells and their corresponding control cells subcutaneously into nude mice. Tumor sizes were measured every 7 days. HCT116/ control cells generated the mean tumor volume of 773.3 mm^3^, while HCT116/ RPS7 cDNA cells generated a reduced tumor volume of 514.1 mm^3^ at 35 days (Figure 4A and 4B, *p* = 0.032). Tumor sizes formed by LOVO/ RPS7 shRNA and LOVO/ control cells at 35 days were 752.2 mm^3^ and 488.7 mm^3^, respectively (Figure 4C and 4D, *p* = 0.038). In addition, compared with the corresponding controls, the overexpression of RPS7 inhibited, but silencing of RPS7 promoted tumor growth by reducing or increasing the weight of tumors (Figure 4E). The body weights of mice in RPS7-overexpressing or silenced group were slightly lighter than those in their corresponding control groups with no significance, suggesting the injection operation had on harm to the nude mice (Figure 4F).

To confirm whether the expression of RPS7 can up-or down-regulate HIF-1α, GLUT4 and LDHB *in vivo*, we performed IHC analyses on tumor sections from all the experimental groups. As shown in Figure 4G, compared with corresponding control groups, the overexpression of RPS7 decreased the expression of HIF-1α, GLUT4 and LDHB, while the silencing of RPS7 enhanced the expression of HIF-1α, GLUT4 and LDHB *in vivo*. These results *in vivo* demonstrated that RPS7 exhibited anti-tumor activities through suppression of HIF-1α, GLUT4 and LDHB in colorectal cancer.

### The expression of RPS7, HIF-1α, GLUT4 and LDHB in CRC and ANT

**Figure 5:**
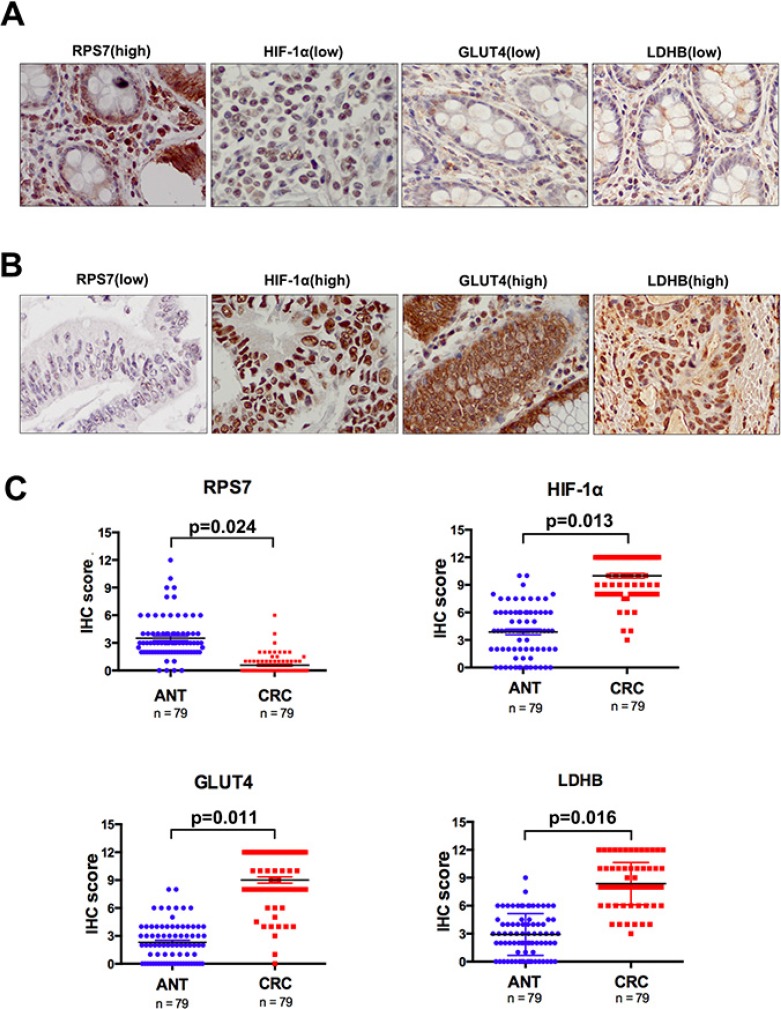
Immunohistochemical analyses of RPS7, HIF-1α, GLUT4 and LDHB expression Representative images from tissue microarray stained for RPS7 and HIF-1α. (**A**) positive expression of RPS7 in nuclei was correlated with negative expression of HIF-1α, GLUT4 and LDHB in the same core of colorectal cancer (× 400). (**B**) negative nuclear accumulation of RPS7 was correlated with positive expression of HIF-1α, GLUT4 and LDHB in the same core of colorectal cancer (× 400). (**C**) the IHC scores of RPS7, HIF-1α, GLUT4 and LDHB in tissue samples of colorectal cancer and corresponding adjacent normal tissues.

We performed IHC and scored the results in TMA consisting of 79 patients with stage I-III colorectal cancer and corresponding adjacent normal tissues (ANT) to test the expression status of RPS7, HIF-1α, GLUT4 and LDHB. We found that the nuclear score for RPS7 was negatively correlated with HIF-1α, GLUT4 or LDHB staining in tumor tissues (*p* < 0.05), evidenced by the representative images showing the high expression of RPS7 corresponding to the low expressions of HIF-1α, GLUT4 or LDHB in same tissues (Figure 5A), or vice versa (Figure 5B). The expressions among HIF-1α, GLUT4 and LDHB were positively correlated (*p* = 0.028). We also found that the expression level of RPS7 in CRC was significantly lower than that in ANT (*p* < 0.05) (Figure 5C and Table 1). However, the IHC scores of HIF-1α, GLUT4 and LDHB were remarkably higher in CRC than those in ANT (*p* < 0.05) (Figure 5C and Table 1).

**Table 1:** Association of RPS7, HIF-1α, GLUT4 and LDHB expressions with CRC TNM stage

	ANT *N* (%)	Stage I *N* (%)	Stage II *N* (%)	Stage III *N* (%)
**Number**	79	6	27	46
**RPS7 expression**				
No staining	3 (3.8)	5 (5.8)	19 (70.4)	31 (67.4)
Weak staining	47 (59.2)	1 (94.2)	8 (29.6)	15 (32.6)
Intermediate staining	23 (29.1)	0 (0)	0 (0)	0 (0)
Strong staining	6 (7.6)	0 (0)	0 (0)	0 (0)
**IHC score**				
Mean ± SE	3.51 ± 2.23	0.17 ± 0.37	0.52 ± 0.69	0.66 ± 1.22
**P^a^**		< 0.01	< 0.01	< 0.01
**P^b^**	< 0.01			
**HIF-1α expression**				
No staining	13 (16.5)	0 (0)	0 (0)	0 (0)
Weak staining	42 (53.2)	2 (33.3)	0 (0)	0 (0)
Intermediate staining	22 (27.8)	4 (66.7)	9 (33.3)	7 (15.2)
Strong staining	2 (2.5)	0 (0)	18 (66.7)	39 (84.8)
**IHC score**				
Mean ± SE	3.88 ± 2.70	10.17 ± 3.29	10.48 ± 1.79	9.67 ± 2.34
**P^a^**		= 0.015	< 0.01	< 0.01
**P^b^**	< 0.01			
**GLUT4 expression**				
No staining	23 (29.1)	0(0)	0 (0)	0 (0)
Weak staining	46 (58.2)	0(0)	4 (14.8)	2 (4.3)
Intermediate staining	10 (12.7)	2 (33.3)	4 (14.8)	19 (41.3)
Strong staining	0 (0)	4 (66.7)	19 (70.4)	25 (54.4)
**IHC score**				
Mean ± SE	2.3 ± 2.05	10 ± 1.63	9.17 ± 2.7	8.78 ± 3.11
**P^a^**		= 0.06	< 0.01	< 0.01
**P^b^**	< 0.01			
**LDHB expression**				
No staining	18 (22.8)	0(0)	0 (0)	0 (0)
Weak staining	39 (49.4)	0(0)	4 (14.8)	3 (6.5)
Intermediate staining	21 (26.6)	4 (66.7)	5 (18.5)	20 (43.5)
Strong staining	1 (1.3)	2 (33.3)	18 (66.7)	23 (50)
**IHC score**				
Mean ± SE	2.91 ± 2.22	8.17 ± 1.21	7.85 ± 2.45	8.7 ± 2.18
**P^a^**		= 0.04	< 0.01	< 0.01
**P^b^**	< 0.01			

a Kruskal-Wallis Test for IHC score of protein expression between adjacent normal tissue (ANT) and corresponding patients with stage I, II, III, respectively;

b Kruskal-Wallis Test for IHC score of protein expression between ANT and all patients;

Abbreviations: ANT, adjacent normal tissue IIHC, immunohistochemical staining; SE, standard error;

### Correlations between the clinical pathologic characteristics and the expression of RPS7, HIF-1α, GLUT4 or LDHB in CRC patients

We analyzed the associations between the expression levels of RPS7, HIF-1α, GLUT4 or LDHB and the patients’ clinical pathologic characteristics. As shown in Table 2, we found that in CRC the expression level of RPS7 differed in tissues with varied T stages (*p* = 0.036), negatively related with T stage (*p* = 0.025). The expression of HIF-1α tended to be positively related with T stage, though not reaching statistical significance (*p* = 0.36). Meanwhile, we found that the expression level of GLUT4 was significantly different in CRC patients with varied differentiation grades (*p* = 0.047) and N stages (*p* = 0.047).

**Table 2:** Association of RPS7, HIF-1α, GLUT4 and LDHB expressions with patient clinical pathologic characteristics

Characteristics	RPS7 N W I S	HIF-1α N W I S	GLUT4 N W I S	LDHB N W I S
**Grade**				
I-II	39 20 0 0	0 3 15 41	0 6 21 32	0 5 34 20
III	13 7 0 0	0 0 0 20	0 0 5 15	0 2 7 11
Total	52 27 0 0	0 3 15 61	0 6 26 47	0 7 41 31
**P^a^**	0.35	0.94	0.047*	0.65
**T stage**				
1–2	9 10 0 0	0 2 6 11	0 2 7 10	0 1 10 8
3–4	43 16 1 0	0 1 19 40	0 3 20 37	0 5 10 45
Total	52 16 1 0	0 3 25 51	0 5 27 47	0 6 20 53
**P^a^**	0.036*	0.36	0.22	0.73
***N* stage**				
0	21 11 0 0	0 1 8 23	0 4 11 17	0 4 18 10
1	17 7 0 0	0 2 7 15	0 1 11 12	0 2 13 9
2	14 9 0 0	0 2 4 17	0 2 8 13	0 1 7 15
Total	52 27 0 0	0 5 19 55	0 7 30 42	0 7 38 34
**P^a^**	0.50	0.25	0.047*	0.65

a Kruskal-Wallis H Test for IHC score of protein expression between subgroups;

* Statistical significance (*P* < 0.05);

Abbreviations: ANT, adjacent normal tissue; N, no staining; W, weak staining; I, intermediate staining; S, strong staining; T, tumor; N, lymph node.

### RPS7 and HIF-1α predict prognosis in CRC patients

**Figure 6:**
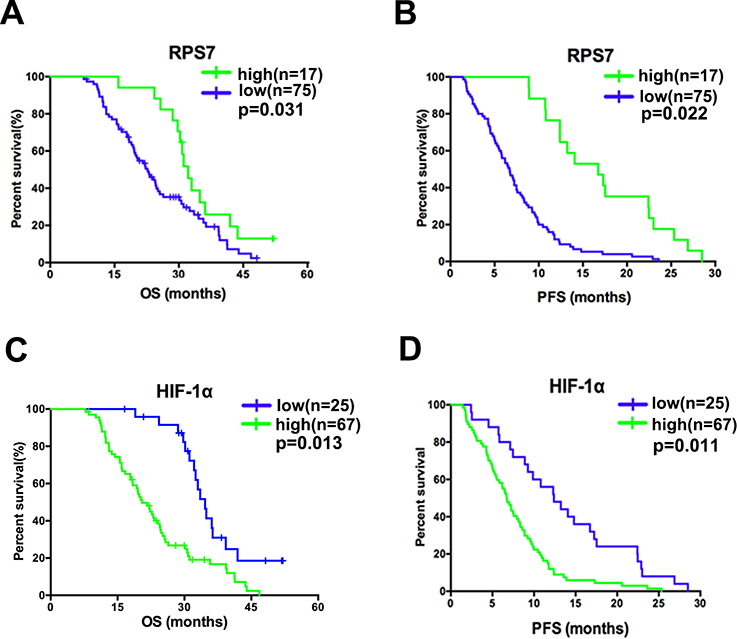
Correlation in advanced colorectal cancer and the associations of the molecules with patients’ survival (**A**) favorable overall survival (*p* = 0.031) and progression-free survival (*p* = 0.022) were associated with positive nuclear accumulation of RPS7. (**B**) poorer overall survival (*p* = 0.013) and progression-free survival (*p* = 0.011) were associated with positive nuclear accumulation of HIF-1α.

To confirm the relationship between the expression level of RPS7 or HIF-1α with the survival of CRC patients, we perform IHC in 92 patients with IV stage disease. The result showed that nuclear accumulation of RPS7 was significantly associated with good overall survival (17 of 92 patients, or 18.5%; *p* = 0.031) and progression-free survival (17 of 92 patients, or 18.5%; *p* = 0.022; Figure 6A and 6B). Strong staining for HIF-1α was significantly associated with poor overall survival (67 of 92 patients, or 72.8%; *p* = 0.013) and progression-free survival (67 of 92 patients, or 72.8%; *p* = 0.011; Figure 6C and 6D). These data suggested that the expression of RPS7 and HIF-1α have the potential to predict the prognosis of patients with advanced colorectal cancer.

## DISCUSSION

Unlimited growth and reprogramed metabolism are two hallmarks of cancer and play important role in cancer progression [19]. For years, great efforts have been made to reverse the unlimited growth and altered metabolism of cancer cells to prohibit tumorigenesis and tumor progression. In this study, we found that RPS7 significantly inhibited the growth, proliferation and glycolysis of CRC both *in vitro* and *in vivo*, which was in correspondence with our previous study indicating RPS7 to be a tumor suppressor [18]. Thus, our results uncovered a potential mechanism by which RPS7 inhibits the growth, proliferation and glycolysis of CRC through the down-regulation of HIF-1α.

It has been found that aberrant promoter hypermethylation of RPS7 in CRC tissues compared with adjacent normal control tissues [32], suggesting RPS7 to be a potential diagnostic marker and therapeutic target of CRC. Furthermore, drug zebalarine inhibits the activity of cancer by stabilizing p53 through RPS7-MDM2 pathways in CRC [33]. These studies indicated that RPS7 has multi-roles in the tumorigenesis and progression in cancers, including colorectal cancer.

HIF-1α was previously found to promote glycolysis and produce lactate by inducing the expression of target genes involved in glucose uptake (glucose transporters) and glycolysis (glycolytic enzymes) [34], including glucose transporter 4 (GLUT4) and lactate dehydrogenase (LDH), an enzyme composed of two subunits LDHA and LDHB [35, 36]. Recently, the study in hepatocellular carcinoma (HCC) demonstrated that the fluorine-18 fluorodeoxyglucose (FDG) uptake level is positively correlated with HIF-1α expression and the expression of HIF-1α showed stronger positive relation with glucose transporter 4 (GLUT4) expression, compared with GLUT2 and GLUT3 [37]. The expression levels of HIF-1α and LDHB were found to be increased in high-grade gliomas compared with low-grade gliomas [38]. In our study, we found that the overexpression of RPS7 decreased the expression level of HIF-1α and further suppressed the expression of GLUT4 and LDHB, leading to the inhibition of growth, proliferation and glycolysis of CRC *in vitro* and *in vivo*. Meanwhile, the overexpression of HIF-1α can rescue the anti-tumor effect of RPS7, suggesting HIF-1α to be a potential target gene of RPS7.

Moreover, we showed that the high expression of RPS7 and low-expression of HIF-1α were significantly associated with good progression-free and/or overall survivals in stage IV CRC patients, which may be used as potential markers for diagnosis and clinical treatment of advanced colorectal cancer. Taken together, our data revealed the negative correlation between RPS7 and HIF-1α, and they modulate the glycolysis of colorectal cancer through molecules including GLUT4 and LDHB, which may represent novel prognostic markers and/or therapeutic targets for colorectal cancer.

## MATERIALS AND METHODS

### Cell lines and cell culture

The established human colorectal cancer cell lines RKO, HCT116, SW620, HT29 and LOVO were obtained from American Type Culture Collection (ATCC). All cells were maintained in Dulbecco's modified Eagle's medium (DMEM, HyClone, Thermo Scientific, USA) supplemented with 10% fetal bovine serum (Gibco, Life technologies, USA), 100 U/ml penicillin (Biowest, Nuaillé, France), and 100 U/ml streptomycin (Biowest, Nuaillé, France) and incubated at 37°C in a humidified atmosphere with 5% CO_2_.

### Plasmids construction and viral infection

The recombinant plasmid pENTER-RPS7, containing human full cDNA sequence of RPS7, was purchased from Vigene Biosciences (Jinan, China), and then the cDNA sequence of RPS7 was subcloned into lentivirus vector pCDH-CMV-MCS-EF1-PURO, generating the recombitant plasmid pCDH/RPS7 cDNA. The DNA oligonucleotides used to generate short hairpin RNA (shRNA) against the open reading frame of RPS7 mRNA (positions 167–189) were 5-TCGGAAAGCTATCATAATCTTTA-3. The recombinant plasmid, pLKO/ shPRS7, was generated according to the previously reported method [18]. The control vector was similarly constructed by directly inserting oligonucleotides encoding short hairpin RNA against green fluorescence protein mRNA (shGFP) into pLKO.1 vector.

Lentivirus carrying RPS7 cDNA or shRNA were generated and harvested as described previously [18]. Briefly, the cells were infected twice for a total of 4 days (2 days for each infection) and the positive clones were selected with puromycin (200 ng/mL) for 7–10 days. Control cell lines were generated by infection with viruses containing the empty vector following the same protocol.

### Real-time PCR

Total RNA from 3 × 10^6^ cells for each cell line was isolated by using Trizol reagent (Invitrogen, Carlsbad, CA). All RNAs were then reversely transcribed into cDNAs that were suitable for real-time PCR analysis using the ExScript RT-PCR kit (TaKaRa, Japan). Oligonucleotide primers for RPS7 were 5′-GTCGTCTTTATCGCTCAGAG-3′ (Forward primer) and 5′-TGTCAGAGTACGGCTCCTG-3′ (Reverse primers). Oligonucleotide primers for HIF-1α were 5′-GAACGTCGAAAAGAAAAGTCT-3′ (Forward primer) and 5′-CCTTATCAAGATGCGAACTCA-3′ (Reverse primers). Oligonucleotide primers for GAPDH were 5′-GGCCTCCAAGGAGTAAGACC-3′ (forward primer) and 5′-CAAGGGGTCTACATGGCAAC-3′ (reverse primers). All amplifications and detections were carried out in the Applied Biosystems Prism 7900 system (Applied Biosystems, Foster City, CA) using the ExScript Sybr green QPCR kit (TaKaRa) and the following program: 95°C for 10 s, one cycle; 95°C for 5 s, 62°C for 31 s, 40 cycles; followed by a 30-min melting curve collection to verify the primer dimers. Statistical analysis was performed using the 2^−ΔΔCT^ relative quantification method.

### CCK-8 proliferation assay

Cell proliferation was determined by CCK-8 assays by using CCK-8 reagents (Dojindo, Japan) and performed according to the manufacturer's protocol. Cells were seeded in 96-well plates (2 × 10^3^ cells per well), 10 μl CCK-8 solution were added to each well at 0, 2, 4, 6, 8 and 10 days, and the plates were incubated at 37°C in 5% CO_2_ for 1 h. The absorbance of each sample was measured at a wavelength of 450 nm using a microplate reader.

### Colony formation assay

1 × 10^3^ cells were seeded in six-well plates at a single cell density and the fresh medium was added to allow cells to grow for at least one week. The colonies with more than 50 cells were counted after staining with gentian violet (Solarbio).

### Glycolysis analysis

Glucose Uptake Colorimetric Assay Kit (Biovision, USA) and Lactate Colorimetric Assay Kit (Biovision, USA) were purchased to examine the glycolysis process in colorectal cancer cells according to the manufacturer's protocol.

### Western blot analysis

Western blot analysis was performed to determine the expression levels of various proteins in cells. Cells were harvested, washed with cold 1 × PBS, and lysed with RIPA lysis buffer (Beyotime) for 30 min on ice, then centrifuged at 12,000 *g* for 15 min at 4°C. The total protein concentration was determined by BCA protein assay kit (Beyotime). Equal amounts (30 μg per load) of protein samples were subjected to SDS-PAGE electrophoresis and transferred on to polyvinylidene fluoride (PVDF) membranes (Millipore). The blots were blocked in 10% non-fat milk, and incubated with primary antibodies, followed by incubation with secondary antibodies conjugated with horseradish peroxidase (HRP). The protein bands were developed with the chemiluminescent reagents (Millipore). Antibodies to RPS7, HIF-1α, GLUT4 and LDHB were from ProteintechTM. The antibody to β-Actin was purchased from Sigma-Aldrich.

### Animal assays

The HCT116 or LOVO cells stably transfected with RPS7 cDNA or shRNA and their corresponding controls by retroviral infection were used for animal assay. To generate tumor growth *in vivo*, 5 × 10^6^ cells of each cell line were subcutaneously injected into 4- to 6-week-old BALB/c athymic nude mice (Department of Laboratory Animal, Fudan University). The animal experiments were approved by the Institutional Animal Care and Use Committee of Fudan University and performed following Institutional Guidelines and Protocols. Each cell line was bilaterally injected into five mice, for a total of 10 injections. The longest diameter “a” and the shortest diameter “b” of tumors were measured and the tumor volume was calculated with the use of the following formula: tumor volume (in mm3) = a × b^2^ × 0.52, where 0.52 is a constant to calculate the volume of an ellipsoid. When a tumor reached 1.0–1.5 cm in diameter, the mouse was sacrificed and the tumors were weighed. Three tumors per cell line were excised, fixed in 10% formalin overnight, and subjected to routine histological examination by investigators who were blinded to the tumor status. Animal assays were repeated twice.

### Patient information

In this study, the tissue microarray (TMA) slides included colorectal cancer tissue samples and corresponding adjacent normal tissue (ANT) samples from 79 patients, who were staged based on the 7th edition of the AJCC Cancer Staging Manual and graded based on WHO criteria. The patients had undergone radical surgery at Fudan University Shanghai Cancer Center (FUSCC) between January 2009 and August 2009. Each case was assigned as an information consent form and was approved by Ethics Committee of FUSCC. The mean age of the 79 patients was 55.39 years (ranging from 30 to 77 years) (Table 3). Regarding to surgical disease stage, 6 (7.6%) patients were stage I disease, 27 (34.2%) were stage II disease and 46 (58.2%) were stage III disease. There were 2 (2.5%) patients with well-differentiated grade, 57 (72.2%) patients with moderate-differentiated grade and 20 (25.3%) patients with poor-differentiated grade. Among these patients, 64 (81%) received adjuvant chemotherapy and 31 (39.2%) received radiotherapy.

**Table 3:** The characteristics of CRC patients in TMA

Characteristic	*N* (%)
**Age at diagnosis (year)**	
Mean ± SD	55.39 ± 11.35
**Gender**	
Male	42 (53.2)
Female	37 (46.8)
**Primary site**	
Rectum	46 (58.2)
Colon	33 (41.8)
**TNM stage**	
I	6 (7.6)
II	27 (34.2)
III	46 (58.2)
**Pathology grading**	
Well-differentiated	2 (2.5)
Moderate-differentiated	57 (72.2)
Poor-differentiated	20 (25.3)
**Histotype**	
Adenocarcinoma	74 (93.7)
Mucinous	5 (6.3)
**Receive chemotherapy**	
Yes	64 (81.0)
No	25 (19.0)
**Receive radiotherapy**	
Yes	31 (39.2)
No	48 (60.8)

Abbreviations: CRC, colorectal cancer; TMA, the tissue microarray; N, number of patients; SD, standard deviation.

Further, we explored whether the expression levels of RPS7 and HIF-1α influence the survival of CRC patients. So we performed Immunohistochemical staining (IHC) to test the expression level of RPS7 and HIF-1α in 92 CRC patients with IV stage disease, who received 5-fluoropyrimidine, leucovorin, and oxaliplatin (FOLFOX) or capecitabine and oxaliplatin (XELOX) as first-line chemotherapy between December 2006 to April 2011 in FUSCC with complete follow-up information. The mean age of these 92 patients was 56.04 years (ranging from 34 to 75 years) (Table 4), 56 (60.9%) patients being male and 36 (39.1%) patients being female. Among them, there were 54 (58.7%) patients with primary cancer located at rectum and the rest were located at colon. 33 (35.9%) patients suffered multiple organs metastasis at diagnosis, and the other 59 (64.1%) patients beard single organ metastasis. Besides, synchrony metastasis occurred in 63 (68.5%) patients. The mean follow-up interval was 26.76 months (ranging from 2.53 to 58.10 months). Until the last follow-up, all the patients suffered progressive disease (PD) for the first-line chemotherapy, and 75 (82.4%) patients were dead. The mean progression-free survival (PFS) was 9.19 months (ranging from 1.43 to 28.50 months) and the mean overall survival (OS) was 25.07 months (ranging from 7.83 to 52.10 months).

**Table 4:** The characteristics of IV stage CRC patients receiving FOLFOX/XELOX as first-line chemotherapy

Characteristic	*N* (%)
**Age at diagnosis (year)**	
Mean ± SD	56.04 ± 9.51
**Gender**	
Male	56 (60.9)
Female	36 (39.1)
**Primary site**	
Rectum	54 (58.7)
Colon	38 (41.3)
**Number of metastatic organs**	
Single	59 (64.1)
Multiple	33 (35.9)
**Time of metastasis**	
Heterochrony	29 (31.5)
Synchrony	63 (68.5)

Abbreviations: FOLFOX, 5-fluoropyrimidine, leucovorin and oxaliplatin; XELOX, capecitabine and oxaliplatin; SD, standard deviation

### Immunohistochemical staining

The tissue microarray used in this study was consisted of core samples from 79 colorectal cancer tissues and corresponding ANT as described above. The TMA was made technically according to the previously published method [39]. The expressions of RPS7 and HIF-1α were detected by IHC. Antibodies used to detect RPS7 and HIF-1α were described as above. The paraffin-embedded sections were pretreated and stained with antibodies by using the previously reported method [18, 39]. The secondary antibodies against mouse or rabbit IgG were supplied in an IHC kit (#CW2069) from Beijing CoWin Bioscience Co. Ltd (Beijing, China). Results were separately judged, evaluated, and scored by two pathologists (Drs. Bin Chang and Xiaoyu Tu) without knowing the patients’ information. The expressions of RPS7 and HIF-1α were determined based on the percentage of positive cancer cells and the staining intensity. The percentage of positive cells (PPC) was divided into five levels as follows: 0 = < 5% of positive cells, 1 = 5 ∼ 25%, 2 = 25 ∼ 50%, 3 = 50 ∼ 75% and 4 = > 75% of positive cells. The intensity of staining (IS) was classified as 0 = no staining, 1 = weak staining (light yellow), 2 = moderate staining (yellow brown), and 3 = strong (dark brown). As for the negative control, the primary antibody was replaced with PBS. The IHC score of protein expression was determined by multiplying PPC and IS, which was divided as 0 for no staining, 1 ∼ 4 for weak staining, 4 ∼ 8 for intermediate staining, and 8 ∼ 12 for strong staining. For clinical pathologic association analysis of TMA, the final score was graded as “low” for < 6 and “high” for ≥ 6. While the expression level of RPS7 was quite low in stage IV CRC patients, we divided them into two groups. One group was “low” for score 0, and the other group was “high” for scores ≥ 1.

### Statistical analysis

The SPSS software (version 18.0) was used for statistical analysis. Student's *t*-test or ANOVA was used to compare quantitative data, and Chi-square test or Kruskal-Wallis Test was used to test qualitative data. The overall survival and disease free survival curves were plotted by the Kaplan-Meier method and statistically analyzed by log-rank test. *P* < 0.05 (two tailed) was considered statistically significant.
